# Sternal Intraosseous Devices: Review of the Literature

**DOI:** 10.5811/westjem.2020.12.48939

**Published:** 2021-03-24

**Authors:** Jared A. Laney, Jonathan Friedman, Andrew D. Fisher

**Affiliations:** * Texas A&M University College of Medicine, Bryan, Texas; † Washington County Ambulance District, Mineral Point, Missouri; ‡ Medical Command, Texas Army National Guard, Austin, Texas; § University of New Mexico School of Medicine, Department of Surgery, Albuquerque, New Mexico

## Abstract

**Introduction:**

The intraosseous (IO) route is one of the primary means of vascular access in critically ill and injured patients. The most common sites used are the proximal humerus, proximal tibia, and sternum. Sternal IO placement remains an often-overlooked option in emergency and prehospital medicine. Due to the conflicts in Afghanistan and Iraq the use of sternal IOs have increased.

**Methods:**

The authors conducted a limited review, searching PubMed and Google Scholar databases for “sternal IO,” “sternal intraosseous,” and “intraosseous” without specific date limitations. A total of 47 articles were included in this review.

**Results:**

Sternal IOs are currently FDA approved for ages 12 and older. Sternal IO access offers several anatomical, pharmacokinetic, hemodynamic, and logistical advantages over peripheral intravenous and other IO points of access. Sternal IO use carries many of the same risks and limitations as the humeral and tibial sites. Sternal IO gravity flow rates are sufficient for transfusing blood and resuscitation. In addition, studies demonstrated they are safe during active CPR.

**Conclusion:**

The sternal IO route remains underutilized in civilian settings. When considering IO vascular access in adults or older children, medical providers should consider the sternum as the recommended IO access, particularly if the user is a novice with IO devices, increased flow rates are required, the patient has extremity trauma, or administration of a lipid soluble drug is anticipated.

## INTRODUCTION

Early vascular access is a key step in providing care for the severely sick and injured.^1^ When intravenous (IV) access is difficult, the use of intraosseous (IO) needles offers the ability for rapid vascular access.^2^ Success of peripheral IV (PIV) access varies from 34–75%, and success is less likely with additional attempts.^3^ Any fluids or medications that can be administered through an IV can also be given IO, including blood products and IV contrast agents.^2^,^4^–^6^ Intraosseous access is now supported for use in Pediatric Advanced Life Support, Advanced Cardiac Life Support, and Advanced Trauma Life Support.^1^,^4^ As this access is vital in emergent cases, a review of the literature is warranted to keep apace of current trends and supports.

Building on preliminary work by Drinker et al. in 1916 and Arnold Josefson in 1934, the IO route for vascular access came to the forefront based on the studies by Tocantins and O’Neill.^3^,^7^–^11^ They refined the procedure over the years with studies on human patients, most often using the sternum, the distal femur, and the proximal tibia.^8^ The military used the sternal IO route during World War II due to the ease of use and the large volumes that could be infused.^3^,^8^,^12^ However, as disposable polyvinyl chloride IV catheters were introduced in the 1950s, IO use became less frequent.^3^,^13^–^14^ Prior to the development of the disposable polyvinyl chloride IV catheters, metal trocars were used, which often became dislodged and caused thrombophlebitis and skin infections.^3^ There were also concerns pertaining to IOs causing osteomyelitis and marrow embolization.^3^ After several decades, there was renewed interest in pediatric IO, which led to regular use by the 1980s and later inclusion in pediatric resuscitation guidelines in 1985.^2^,^13^,^15^–^16^

Treatment protocols suggest IO as a primary access option in select situations.^17^–^18^ However, it is more traditionally used as an option for vascular access, after failed PIV attempts.^8^,^19^ There are several reasons for the device-selection priority recommendations listed in the guideline literature. While a PIV can be left in place for several days, IO devices are not recommended to be left in place for more than 24 hours.^8^,^20^–^21^ Additionally, IO is significantly more expensive than PIV, approximately $80–$120 per use vs $1–$2 per use for PIV.^22^–^23^ This is in contrast to US military personnel who may use IO or PIV as first-line vascular access in combat.^15^

There are three primary IO sites in use today: the proximal tibia; the proximal humerus; and the sternum.^8^ However, other sites, which include the distal radius and ulna, iliac crest, and medial malleolus, may be used.^24^ There are multiple IO devices on the market today, but the most popular in the literature are the EZ-IO for the proximal tibia and proximal humerus (Teleflex, Inc, Wayne, PA) and the First Access for Shock and Trauma (FAST1) devices for the sternum (Teleflex, Inc, Wayne, PA).^2^,^4^,^8^,^25^ In the early 2000s with the development of the FAST1 and conflicts in Afghanistan and Iraq, the sternum became a popular location for military IO device placement.^15^,^22^,^26^–^27^ It has since become more widespread in the civilian setting as well.^28^–^29^

## METHODS

We searched PubMed and Google Scholar for articles using a combination of the keywords “sternal IO,” “sternal intraosseous,” and “intraosseous” without specific date limitations. We evaluated case reports and series, retrospective and prospective studies, systematic reviews and meta-analyses, and other narrative reviews. We also reviewed guidelines and supporting citations of included articles. The literature search was restricted to studies published in English. When available, systematic reviews and meta-analyses were preferentially selected. These were followed sequentially by randomized controlled trials, prospective studies, retrospective studies, case reports, and other narrative reviews when alternate data were not available. A total of 47 articles were selected for inclusion in this narrative review.

## DISCUSSION

As with other IO devices in civilian settings, the sternal IO is indicated when vascular access is necessary, but PIV has failed or is not readily accessible.^19^ Historically, the use of sternal IO in young children has been associated with higher rates of anatomy-related complications such as increased risk of damage to retrosternal structures and low flow due to small marrow reservoir.^3^,^30^ Currently, sternal IOs are only US Food and Drug Administration-approved for patients aged 12 years and older.^32^

### Anatomy

Intraosseous access makes use of several characteristics of mammalian bones. First, their medullary cavities are non-collapsible as a result of both bone hardness as well as spicule formation. Therefore, they are readily accessible, even in profound hemorrhagic or hypovolemic shock. Furthermore, the bone marrow of the medullary spaces is directly connected to the central venous system via the medullary venous channels.^2^ In the manubrium, blood flows from the marrow space into the internal thoracic vein, which drains to the subclavian vein and central vasculature. This stands in contrast to the humeral and tibial routes, which are farther from the central venous system and less direct.^9^ The most common access point for sternal IO devices is the manubrium.^3^ An additional advantage of the sternum, since the marrow cavities of the sternal body and the manubrium seldom communicate, is that both can be cannulated simultaneously.^3^

### Pharmacokinetics

The pharmacokinetics of sternal IO devices have been shown to be equivalent or superior to extremity IOs and PIVs. Using a swine model of traumatic cardiac arrest, Burgert et al. concluded that the pharmacokinetics of epinephrine for humeral IOs and sternal IOs were statistically equivalent to PIV.^19^ In the same study, maximum epinephrine concentration – C(max) – and time to maximum epinephrine concentration – T(max) – were significantly longer in the tibial IO group as compared to the sternal IO, humeral IO, and PIV groups. Hoskins et al. had similar findings in a cardiac arrest with ongoing cardiopulmonary resuscitation (CPR) swine model.^33^ Using dye tracers, co-administered with and used as a surrogate for, epinephrine, C(max) was reached faster in sternal IO vs. tibial IO.^33^ Additionally, they found that the total dose delivered for tibial IO was only 65% of that delivered via sternal IO. When comparing sternal IO vs the central venous (CV) route, sternal IO delivered 85% of the CV-delivered dose. Overall pharmacokinetics were equivalent when comparing sternal IO vs. central venous administration. Vasopressin has also been found to be equivalent when comparing sternal to PIV administration in terms of C(max), T(max), and mean concentration over time.^34^ Burger et al. found that for amiodarone, a lipid soluble medication, C(max) was slowest for tibial IO and equivalent for both sternal IO and PIV.^35^ The authors hypothesized that the lipid-rich marrow in the tibial site resulted in an amiodarone depot, delaying release to the peripheral circulation.

### Hemodynamics

Two similar studies evaluated hemodynamics after hemorrhage and administration of Hextend through a sternal IO compared to an PIV and/or a humeral IO.^36^–^37^ The models were bled 30% of their total blood volume^36^ and 30% total blood volume based on 70 kilograms human,^37^ and then administered 500 milliliters (mL) of Hextend under pressure. The common hemodynamics measured included heart rate, systolic blood pressure, diastolic blood pressure, mean arterial pressure, stroke volume, and cardiac output. Between the devices being evaluated, there were no significant differences among the hemodynamic variables measured. Additionally, the time required to administer the Hextend was not significantly different between the groups.

### Flow Rates

Using fixed and unfixed cadavers, Hammer et al. found higher flow rates and larger flow volumes in sternal IO compared to humeral IO and tibial IO infusions, both under pressure and unpressurized.^38^ Interestingly, flow rates decreased at both the sternal and tibial IO sites after five minutes while flow rates increased at the humeral sites. In a different cadaver study that measured rates and volumes infused during a five-minute 0.9% sodium chloride bolus, there were again greater flow rates and flow volumes with sternal IO vs humeral IO or tibial IO devices.^39^ Additionally, flow was good (fast drip without pressure bag) or very good (continuous flow without pressure bag) in nearly 90% of the sternal IOs. In a 2019 human field study, 31.5% of EZ-IOs demonstrated poor flow (requiring pressurized infusion), whereas none of the FASTResponder lines had poor flow.^28^ Using human volunteers, unpressurized infusion, and two different sternal IO devices, Bjerkvig et al. were able to deliver 450 mL of whole blood in approximately 11 minutes.^4^

### Ease of Use

Many medical providers find IOs easy to use. In a small study, 10 experienced paramedics evaluating a new sternal IO system (FAST1) found sternal IO placement to be easier than PIV.^27^ Time from package opening to fluid flowing through the device averaged just over 90 seconds, and device placement by these novel users was reported as excellent. Another study reported a 95% (18/19) first-time success rate for sternal IO placement among novice sternal IO device users (second- and fourth-year medical students); this was better than their rate for tibial IO and humeral IO devices, which they also had never used before: 91% (20/22) and 77% (17/22), respectively.^38^ Elsewhere, a study found sternal IO placement was achieved with 100% success by the second attempt with a median insertion time for the FASTResponder of 20 seconds.^28^

In cardiac arrest patients, FAST1 deployment by paramedics had a 73% success rate and an average time to placement of 67 seconds.^29^ Another study that compared time to fluid administration among different IO sites found no statistically significant differences between humeral IO, sternal IO, or IV groups.^36^ Similarly, Hammer et al. found no difference in insertion time between the humeral IO, sternal IO, and tibial IO groups in their study.^38^

When comparing two long bone IO devices, the Bone Injection Gun (PerSys Medical, Houston, TX) and the Jamshidi (Cardinal Health, Dublin, OH) to the FAST1, the long bone devices were equivalent to the FAST1 with respect to success rate, user satisfaction, and complication rates. However, the Jamshadi had a faster mean insertion time than the FAST1 (38 vs 62 seconds, *P* = 0.002).^40^ In contrast to the studies above reporting excellent success rates for novice sternal IO device users, a study evaluating 29 emergency medical technician-basic students noted a first-attempt successful placement of only 55.2%; this was despite high rates of correct site identification (96.6%) and a median time to needle deployment of under 30 seconds.^41^

### Pain

The literature comparing pain associated with sternal IO compared to PIV or peripheral IO is sparse. Preliminary work has been done by Montez et al. evaluating the use of lidocaine to mitigate the discomfort during sternal IO and proximal humerus IO infusions.^42^ The primary endpoints of the study were pain scores at five minutes using 300 millimeters mercury (mm Hg) to pressure infuse, then again after 15 and 30 minutes of infusion at 125 mL/hour. The published data do not evaluate the sternal IO and humeral IO groups against each other. However, useful analysis can be made by the reader: the difference between the pain scores recorded for the 40-milligram (mg) lidocaine dose, 3.4/10 at the sternal IO site and 3.5/10 at the humeral IO site, are clearly not clinically significant. In the 60 mg lidocaine dose groups, the pain scores were 1.5/10 (sternal) and 2.2/10 (humeral), similarly displaying no clinical significance in the difference noted. Additional work is needed to more fully characterize the pain experienced during placement of and infusion with sternal IO compared to peripheral IO and PIV.

### Return of Spontaneous Circulation (ROSC)

With regard to ROSC, sternal IO-administered fluids/medications appear to be at least as effective as PIV and other IO routes. In a 2019 swine model of traumatic (hemorrhagic) cardiac arrest, in which epinephrine followed by 500 mL of 5% albumin was administered, no difference in ROSC was noted between tibial IO, humeral IO, sternal IO, and PIV groups.^19^ An earlier hemorrhagic cardiac arrest model similarly found that ROSC timelines, results, and outcomes were equivalent when comparing sternal IO and PIV groups.^43^ However, an additional cardiac arrest model (ventricular fibrillation, no hemorrhage/exsanguination) found that ROSC occurred faster with sternal IO and PIV-administered medications (vasopressin, amiodarone, and epinephrine) as compared to tibial IO-administered medications.^44^

### Advantages of the Sternal Intraosseous Route

As noted above, flow rates have been shown to be greater in sternal IO vs other IO routes. While this may seem counter to Poiseuille’s Law (Q=(πPr^4^)/8ηl) due to the dimensions of the FAST1 and EZ-IO catheters, bone characteristics play a role as well. The sternum is a large, flat bone with a high amount of red marrow.^26^ It also has a cortex that is thinner and more uniform and is less likely to be fractured as compared to the bones of the arms or legs.^26^ Furthermore, because the sternum is a non weight-bearing bone, its density is predicted to be 25% less than the proximal humerus.^5^ Therefore, lower infusion pressures are required and flow rates are higher with a sternal as compared to a humeral route. As an additional anatomic advantage, the sternum can be identified in every shape and size person, to include the morbidly obese.^3^

There are advantages to the sternal IO route when compared to PIV routes. Findlay et al. noted that its central location as well as its readily identifiable placement site serve to reduce clutter as well as the chance of line entanglement and accidental dislodgement—a concern for both PIV and humeral IO/tibial IO sites.^27^ Additionally, the insertion site has minimal overlaying tissue except for in extreme obesity, again making identification straightforward.^35^ Sternal IO devices also have more direct access to central circulation via the venous drainage of the manubrium, as Burgert et al. noted, and may actually benefit from “the hydraulic action of chest compressions” in cases of cardiac arrest.^33^,^35^ Finally, FAST1 IO devices do not require selection of different needle sizes and rely on deployment of a single needle size to the correct depth.^32^ This reduces the possibility for error and increases cognitive offloading of needle selection based on patient size during stressful patient care scenarios.

### Risks and Limitations

#### Contraindications

Contraindications for sternal IO use are similar to other IO sites and include fracture at the insertion site, IO attempt at the same location in the previous 48 hours, hardware in the vicinity of the anticipated IO placement site, compartment syndrome, significant bone disease at the insertion site, and local infection/osteomyelitis history.^2^,^8^ The only added contraindication for sternal IO is a history of sternotomy, due to the potential for decreased blood flow and impaired structural integrity in the area after this procedure.^8^

#### Risks

Risks of IO use in general are few, and serious complications are rarely experienced.^22^ These include infection (including osteomyelitis and mediastinitis), compartment syndrome, fractures, drug/fluid extravasation, skin necrosis, arterial thrombosis, air/fat embolism, perforation of the opposing cortex, and retained foreign body.^8^,^22^ Epiphyseal plate damage can occur from humeral IO and tibial IO, but not sternal IO.^8^ Potential minor complications include pain, difficulty aspirating marrow, device displacement, and slow or stopped infusion.^9^,^20^ As the manubrium bone is fairly thick at approximately 13.30 mm, the risk of excessive penetration is less than 0.0001%.^31^

#### Myths Explained

There are myths and falsehoods associated with sternal IOs and IOs in general. Some believe that IO flow rates are insufficient for mass transfusion in hemorrhaging patients. However, Bjerkvig et al. found gravity flow rates in two different sternal IO devices to be sufficient for resuscitation in human volunteers.^4^ In their retrospective study of over 1000 IO device deployments, Lewis and Wright noted packed red blood cells transfused successfully nearly 2000 times with no clinical or lab evidence of hemolysis.^22^

Another concern is that sternal IO will interfere with ongoing CPR.^28^ Multiple successful swine studies have been conducted with active CPR and concurrent sternal IO fluid administration without issue.^19^,^33^–^35^,^44^ Based on the findings of their swine study, Hoskins et al. recommended that sternal IO be used preferentially for drug delivery over other IO sites when PIV has not been established.^33^ Several recent human field studies have been conducted looking at the use of sternal IO.^15^,^26^,^28^–^29^,^40^ While only Hartholt et al. specifically mention ongoing CPR, it may be reasonably assumed that CPR was ongoing in at least some of the other patients studied as well. There is no note in any of these papers regarding the sternal IO device interfering with the ability to appropriately perform chest compressions for CPR. Additionally, there are no reports in the literature of sternal IO devices interfering with chest compressions. Further study looking specifically at chest compressions with concurrent sternal IO in place is needed to more conclusively comment on the relationship between the two, but the lack of negative reports in the literature thus far is promising.

#### Limitations

As noted above, the FAST devices are only approved for use in patients aged 12 years and older.^31^ There is also some question of device and operator failure rates. Byars noted that 7/41 attempted FAST1 insertions failed because the needle did not deploy as intended. Additionally, two other attempts were abandoned due to extravasation after placement.^29^ However, this was several years ago and it is unclear whether failure was due to operator error or design flaw or whether the manufacturer has since addressed the problem. Another study noted that stylets in 3 of 22 FASTResponder devices failed to completely withdraw into the protective cover, creating a needlestick risk.^45^ Additionally, the FAST devices are designed only for sternal use, while the EZ-IO devices can be used in either the humerus or tibia.

Obesity and its associated comorbidities and care complications are ubiquitous in nearly every setting served by medical providers. These challenges become particularly apparent when this population is critically ill and can make vascular access extremely difficult. While there tends to be less tissue overlying the sternum than other potential IO access sites, in the extremely obese, sternal IO access may be compromised as well.^26^,^46^ This issue can be mitigated to some degree with peripheral IO devices having multiple needle sizes available, but still presents a challenge. Unfortunately, this feature is not available for the FAST devices, a shortcoming that may preclude their use in patients with excessive parasternal tissue.

Although any drug or fluid that can be given via PIV can also be given IO, there is a paucity of data regarding IV contrast given by the IO route. The existing studies report successful IO contrast administration by hand as well as power injection, resulting in high-quality images; however, these studies either exclusively looked at peripheral IO sites/devices or did not specify the type of device used.^47^–^52^ Further investigation into sternal IO administration of contrast agents is warranted. Finally, a 2015 cadaver study by Hammer et al. demonstrated a wide variation in flow rates, a finding that has not been replicated/verified in human field studies or live human model studies.^38^

## CONCLUSION

The sternum offers an easily accessible route for IO delivery of fluids and medications with high rates of successful placement, even among novices, according to most studies.^27^,^38^ Once the needle has been deployed and the device secured, sternal IO may provide reduced risk for line entanglement as compared to PIV and other IO sites, and there is mounting evidence for superior flow rates with sternal IO.^27^–^28^,^38^–^39^ When considering IO vascular access in adults or older children, medical providers should consider the sternum as the recommended IO access, particularly if the user is a novice with IO devices, increased flow rates are required, the patient has extremity trauma, or administration of a lipid soluble drug is anticipated.
